# An Overview of the Temporal Shedding of SARS-CoV-2 RNA in Clinical Specimens

**DOI:** 10.3389/fpubh.2020.00487

**Published:** 2020-08-20

**Authors:** Khrystyna Zhurakivska, Giuseppe Troiano, Giuseppe Pannone, Vito Carlo Alberto Caponio, Lorenzo Lo Muzio

**Affiliations:** Department of Clinical and Experimental Medicine, University of Foggia, Foggia, Italy

**Keywords:** coronavirus 2019, Covid-19, SARS-CoV-2, specimens, pharyngeal swabs, feces, sputum

## Abstract

Coronavirus disease 2019 quickly spread in China and has, since March 2020 become a pandemic, causing hundreds of thousands of deaths worldwide. The causative agent was promptly isolated and named SARS-CoV-2. Scientific efforts are related to identifying the best clinical management of these patients, but also in understanding their infectivity in order to limit the spread of the virus. Aimed at identifying viral RNA in the various compartments of the organism of sick subjects, diagnostic tests are carried out. However, the accuracy of such tests varies depending on the type of specimen used and the time of illness at which they are performed. This review of the literature aims to summarize the preliminary findings reported in studies on Covid-19 testing. The results highlight how the pharyngeal swab is highly sensitive in the first phase of the disease, while in the advanced stages, other specimens should be considered, such as sputum, or even stool to detect SARS-CoV-2. It highlights that most patients already reach the peak of the viral load in the upper airways within the first days of displaying symptoms, which thereafter tend to decrease. This suggests that many patients may already be infectious before symptoms start to appear.

## Introduction

Coronavirus disease 2019 (COVID-19) quickly spread in China was declared a became pandemic in March 2020, causing hundreds of thousands of deaths worldwide. The causative agent, a virus from the coronavirus family, was promptly isolated and named SARS-CoV-2 (1). The characteristics that make this virus highly dangerous for the population are represented by a very high transmission capacity, as well as by its complex interaction with the host's organism which in a variable, but high percentage of cases, can lead to death (2). Transmission through respiratory droplets, indirect contact, as well as airborne transmission of the virus has been confirmed and the diagnosis is made combining clinical, radiographic (chest Computer Tomography), and laboratory evaluations. In particular, the presence of viral RNA in the pharyngeal swab is analyzed using the real-time reverse transcription-polymerase chain reaction (RT-PCR) (3–5). Regarding the molecular targets that can be used for PCR assays, some structural proteins were identified, among which: spike (S), envelope (E), transmembrane (M), helicase (Hel), and nucleocapsid (N). Furthermore, other genes that are required for viral replication, like RNA-dependent RNA polymerase (RdRp), hemagglutinin-esterase (HE), and open reading frames (ORF1ab) may be targeted for virus detection by RT-PCR (4, 6). There are different recommendations among countries regarding the choice of target (4, 7), nevertheless, to obtain a reliable result, at least two molecular targets should be included in the assay (8). The result of RT-PCR, expressed in Cycle threshold (Ct) provides an answer about the presence or absence of the viral RNA and also estimates the viral load in the sample, where the Ct is inversely proportional to the quantity of the viral RNA. Even if so, it seems that positivity diagnosed with RT-PCR is not indicative of the contagiousness of the patient (1).

Scientific efforts at this time are directed on multiple fronts: on the one hand, researchers are studying the best clinical management of infected patients; on the other hand, they are trying to define the infectious aspects of these patients. In particular, it is necessary to understand when the SARS-CoV-2 positive subject is capable of infecting others or when this possibility is greater? In which biological materials is the virus present and in what quantities? How do these values change during the course of the disease? Are they related to the symptoms?

Partial answers to these questions come from an increasing number of studies that have reported the clinical and virological data of patients, observed in various parts of the world. However, these data often relate to a few patients or only focus on some aspects and not others.

This review aims to summarize the findings of the studies published until now regarding the trend of temporal shedding of SARS-CoV-2 RNA in various clinical specimens.

## Materials and Methods

The electronic database PubMed was screened in order to select studies suitable for inclusion in this review. The following strategy of search was used: [(“SARS-CoV-2” OR “2019-nCoV” OR “covid-19”) AND (load OR samples OR specimens)]. In addition, bibliographies of the included studies were read, and suitable references researched separately.

The results were screened by title and abstract, selecting the records fulfilling the following inclusion criteria:

- studies published in English;
- studies reporting data on SARS-CoV2 RNA evaluation in clinical specimens with chronological reference to the illness course.

No restrictions on the study design were applied.

The established exclusion criteria consisted of:

- studies written in languages other than English;
- studies evaluating treatment options;
- non-original studies;
- studies without a clear reference to the onset of the disease (onset of symptoms).

In case of insufficient information after abstract reading, the full-text publication was examined.

The selected papers were full-text evaluated and, if meeting the inclusion criteria, were included in the review.

An *ad hoc* datasheet containing queries was prepared and the following data, if available, was extracted and inserted into the datasheet:

- Author's names;
- number of patients;
- type of specimen analyzed and results of RT-PCR with the corresponding days of illness from symptom onset to which they refer;
- molecular target used in the RT-PCR analysis.

### Qualitative Analysis

The results of the examined specimens reported for every day of patients' illness were collected. If the result of the test was positive, according to the parameters established in the original paper, a “+” was assigned, while a “–” was assigned if the test result was negative.

No distinction was made on the methodology used in the various studies, nor on the unit of measure, only a dichotomous result (+ or –) was reported.

The total percentage of positives and negatives was thus determined day by day, for each type of sample.

### Quantitative Analysis

The cases for which the Ct values of RT-PCR were reported for every single test were included in this analysis. The data were grouped by type of target (i.e., ORF1ab, E, S, RdRp etc.) used for virus RNA detection in every type of specimen. The mean and standard deviation of Ct values were calculated for each day of patients' illness.

### Other Analysis

The descriptive results that could not be included neither in a quantitative nor in a qualitative analysis, were also collected.

## Results

A total of 243 records were found, applying the search strategy on electronic databases. After the title and abstract examination, 25 abstracts fulfilled the inclusion criteria and were selected for a full-text reading. Of these, 21 (7, 9–27) were deemed suitable for inclusion in the review. Generic information about the included studies are reported in Table 1.

**Table 1:** Main characteristics of included studies.

**Author**	**N° of patients**	**Country**	**Investigated specimens**	**N° patients included in quantitative analysis**	**N° patients included in qualitative analysis**
Chen et al. (10)	57	China	Pharyngeal swab, blood, anal swab,	6	6
Chen et al. (11)	42	China	pharyngeal swab, stool, urine	0	0
Holshue et al. (7)	1	USA	Naso-and oropharyngeal swabs, blood, feces, urine	0	1
Kam et al. (13)	2	Singapore	Pharyngeal swab, blood, feces, urine, mother's breast milk	1	1
Kim et al. (12)	2	Korea	Naso- and oropharyngeal swabs, serum, plasma, sputum, feces, urine	2	2
Lan et al. (9)	4	China	Oropharyngeal swabs	0	0
Lescure et al. (14)	5	France	Pharyngeal swab, plasma, feces, urine, conjunctiva	0	5
Liu et al. (27)	12	China	Oropharyngeal swab, Bronchoalveolar lavage, Fluid	6	6
Lo et al. (15)	10	China	Nasopharyngeal swab, sputum, urine, feces	0	0
Pan et al. (16)	82	China	Oropharyngeal swab, sputum, feces, urine	0	2
Qiu et al. (28)	10	China	Vaginal fluids	0	0
To et al. (17)	12	China	Saliva	0	0
To et al. (18)	23	China	Blood, saliva, anal swab, urine	0	0
Wang et al. (19)	205	China	Pharyngeal swab, blood, sputum, nasal swab, bronchoalveolar lavage fluid, Fibrobronchoscope brush biopsy, feces, urine	0	0
Wölfel et al. (23)	9	Germany	Pharyngeal swab, sputum, feces	0	9
Xiao et al. (24)	73	China	Pharyngeal swab, stool	0	0
Yang et al. (26)	213	China	nasal swabs, throat swabs, sputum, bronchoalveolar lavage fluid	0	0
Young et al. (20)	18	Singapore	Nasopharyngeal swab, blood, feces, urine	18	18
Yu et al. (21)	76	China	Nasopharyngeal swab, oropharyngeal swabs, plasma, sputum, nasal swab, urine	0	0
Zhang et al. (22)	15	China	Oral swab, anal swab, blood	0	0
Zou et al. (25)	18	China	Oropharyngeal swab, nasopharyngeal swab	18	18

**Figure 1:**
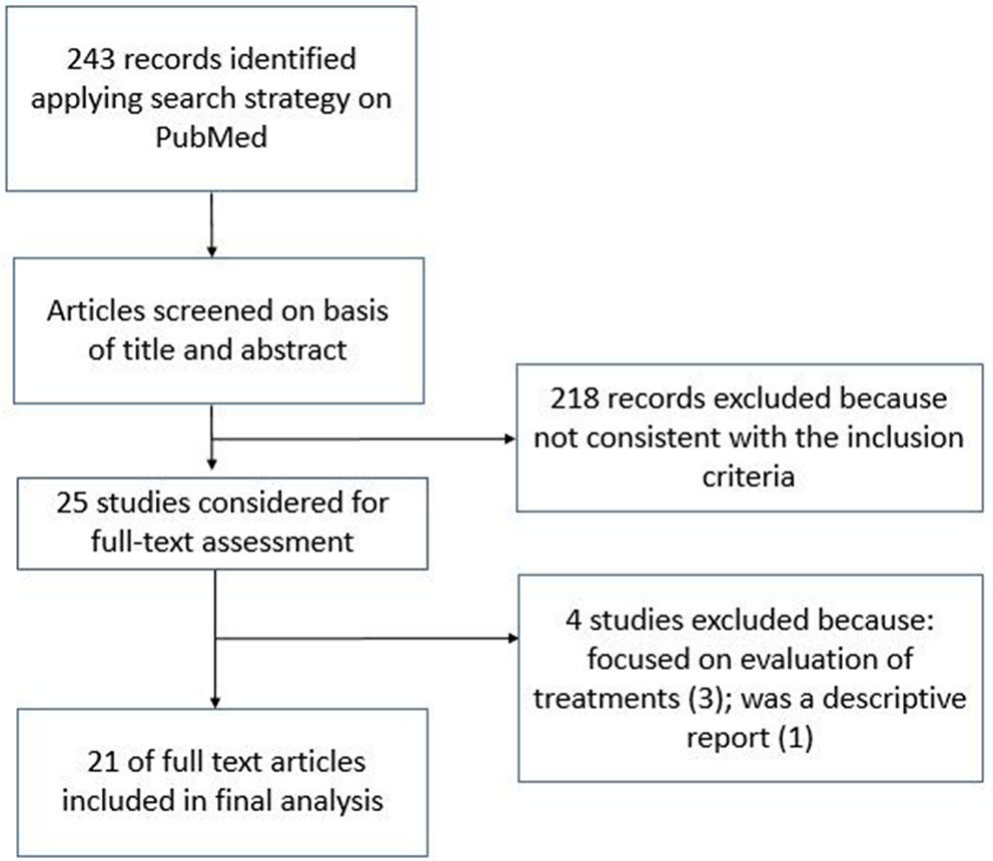
Flowchart representing the selection process of the studies suitable for inclusion.

The discarded articles were focused on the evaluation of some treatments and therefore considered misleading for the purposes of our evaluation (29–31). One study was only a descriptive report and was also excluded (32). A flowchart representing the selection process is reported in Figure 1.

Due to a large variability among studies in methodology and presentation of results, only six studies were included in the quantitative evaluation, while for others a qualitative or discursive consideration was performed.

### Qualitative Results

**Figure 2:**
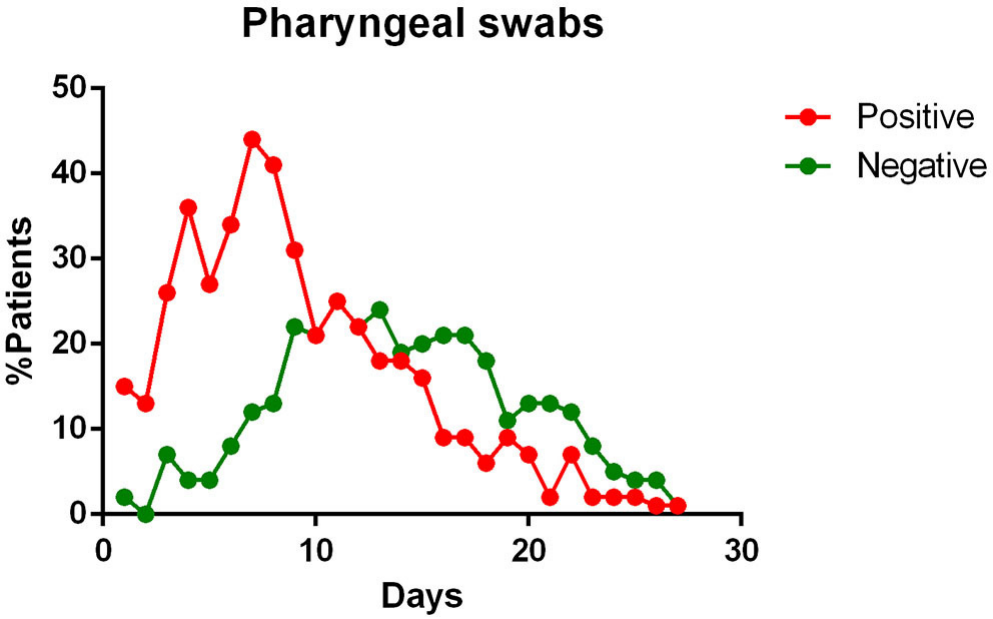
Positivity and negativity rates of Pharyngeal specimens along the time of illness. Each dot represents the percentage of the analyzed specimens that resulted positive or negative on that specific day.

**Figure 3:**
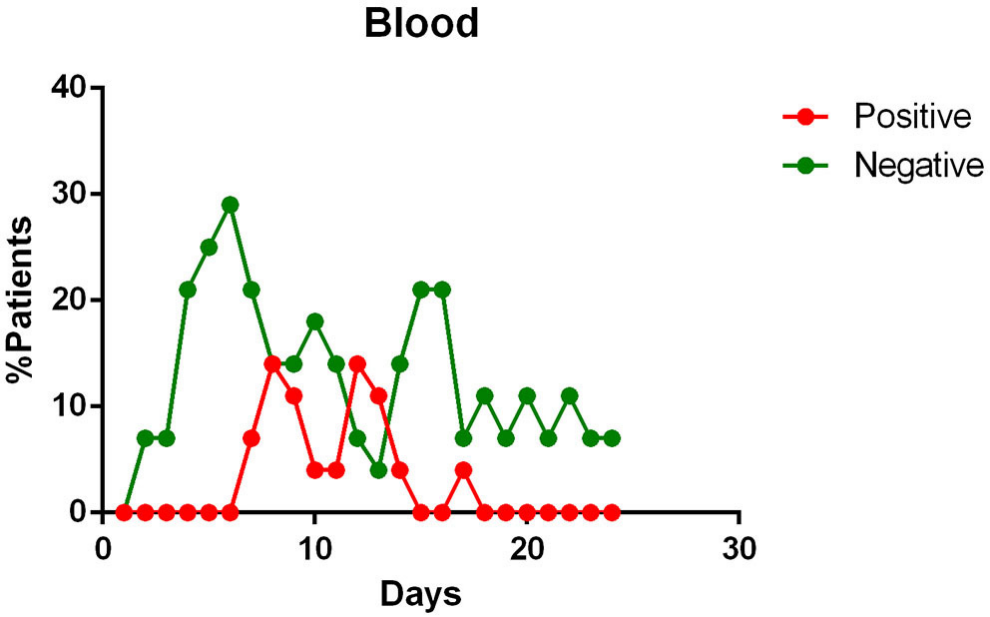
Positivity and negativity rates of Blood specimens along the time of illness. Each dot represents the percentage of the analyzed specimens that resulted positive or negative on that specific day.

**Figure 4:**
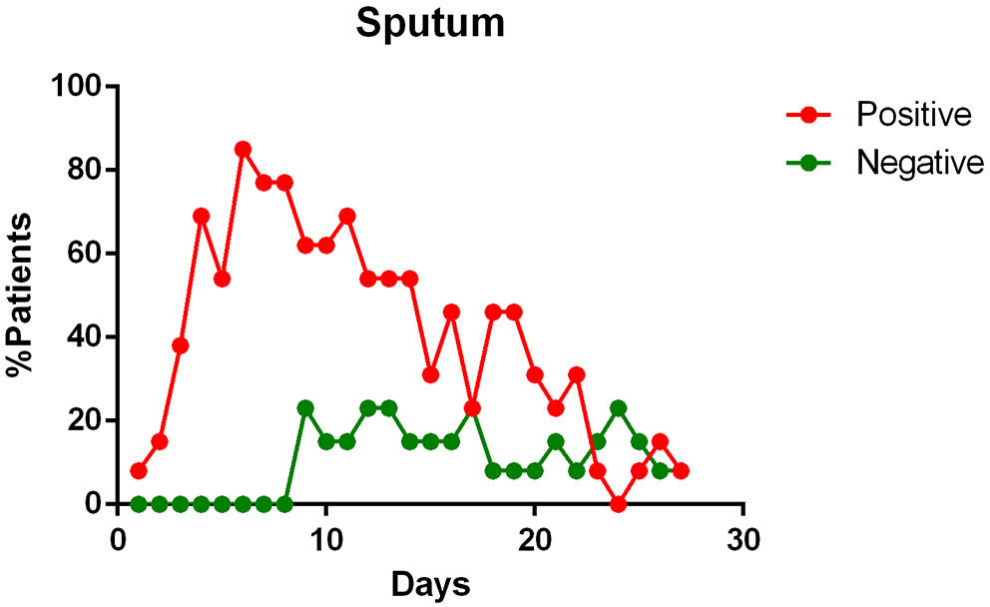
Positivity and negativity rates of Sputum along the time of illness. Each dot represents the percentage of the analyzed specimens that resulted positive or negative on that specific day.

**Figure 5:**
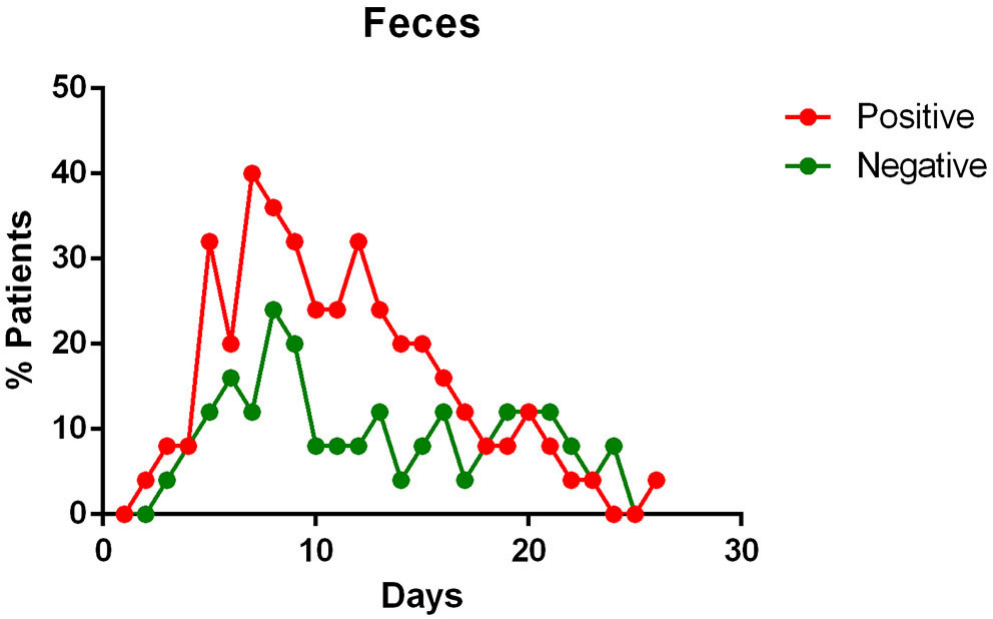
Positivity and negativity rates of Feces along the time of illness. Each dot represents the percentage of the analyzed specimens that resulted positive or negative on that specific day.

**Figure 6:**
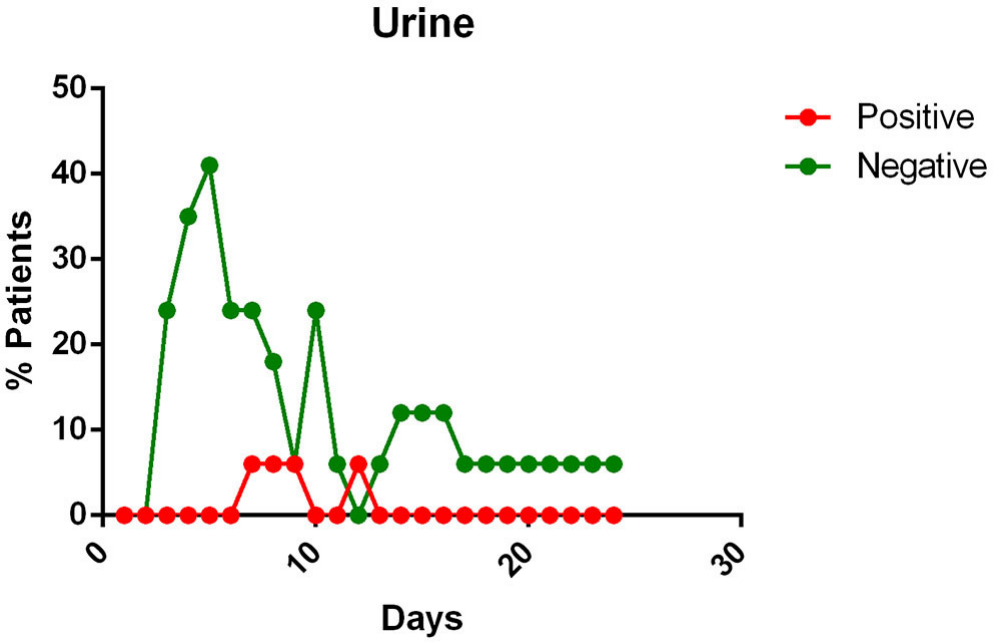
Positivity and negativity rates of Urine along the time if illness. Each dot represents the percentage of the analyzed specimens that resulted positive or negative on that specific day.

In the final qualitative analysis 68 patients were included. Of these, complete temporal data, with reference to the day of illness were available for: pharyngeal specimens in 68 patients; blood specimens in 28 patients; feces samples in 25 patients; urine in 17 patients; sputum in 13 patients. The main findings of data analysis revealed that:

- most patients had a positive Pharyngeal swab result for the first 10 days of illness. After this term, the percentage of patients whose Pharyngeal swab result was negative increased, and then even exceeded the positive ones around day 12 of illness (Figure 2);
- viral RNA was not detected in the blood of most patients. In <15% of patients, viremia was registered in the second week of illness (Figure 3);
- sputum contains viral RNA throughout the duration of the disease (Figure 4);
- the virus is eliminated in the stool of sick patients. Toward the end of the first week of the disease, viral RNA was found in approximately 40% of patients (Figure 5);
- the urine of Covid-19 patients was almost always negative for the presence of the virus (Figure 6).

**Figure 7:**
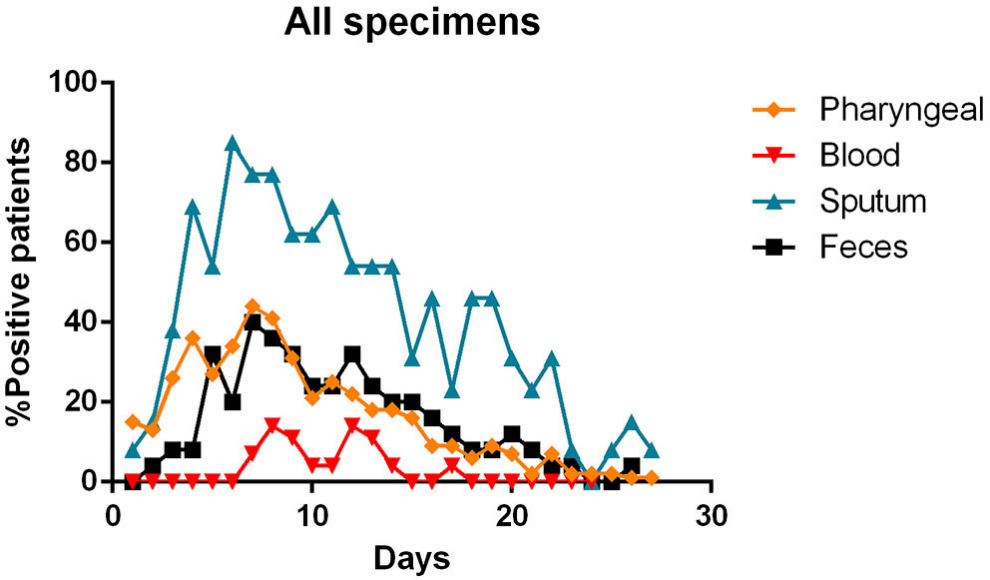
Percentages of positivity observed for the main types of specimens during the illness of included patients. Each dot represents the percentage of the analyzed specimens that resulted positive or negative on that specific day.

The Figure 7 summarizes the percentages of positivity observed for each type of specimen during the patients' illness.

### Quantitative Results

Data related to 51 patients were included in the quantitative analysis. The table included in the Supplementary Material summarizes information about the analyzed specimens, molecular targets used, and the Ct values observed at RT-PCR analysis.

**Figure 8:**
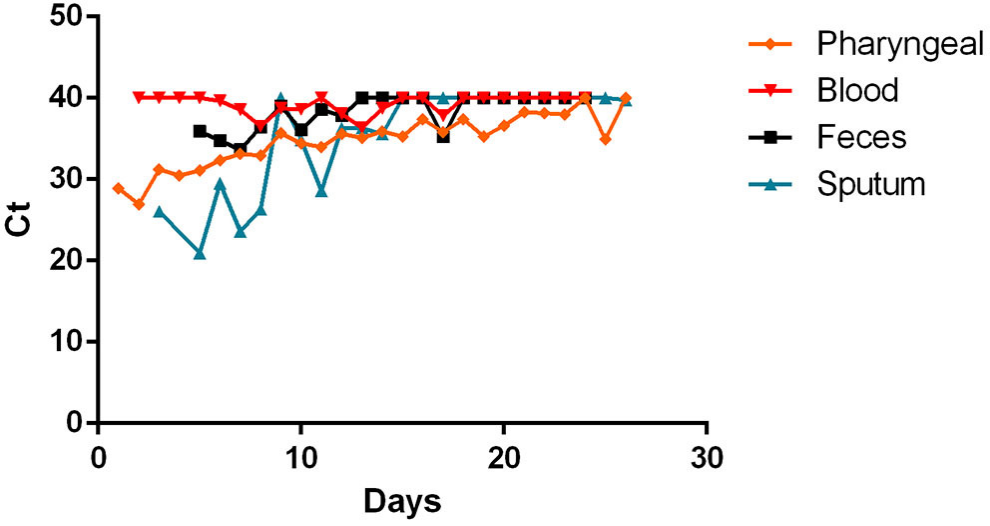
The time course of RT-PCR Ct values in main specimens.

The time course of RT-PCR Ct values related to the most representative specimens are reported in Figure 8 in a cumulative way, regardless of the type of molecular target. The representation of Ct values in specimens, divided by type of molecular target are present in the Supplementary Materials.

*Other results* reported in the included studies which were not considered in the quantitative and qualitative analyses, affirm as follows:

#### Upper Respiratory Samples

Pharyngeal viral load is highest in the early phase of illness (12, 16, 20–23, 25), showing high levels already in the first 24 h from the onset of symptoms (14) with the peak on the 5–6th day of illness (16). According to Wolfel et al., in this period the detection rate was 100%, decreasing substantially after day 5, with a detection rate that more than halves (39.93%) (23). Furthermore, the study of subgenomic messenger RNAs (sgRNA) suggested that the first 5 days of illness are characterized by an active replication of SARS-CoV-2 in the upper respiratory tract, while after the 5th day, no sgRNA was detected in pharyngeal samples (23). In the advanced stage of the disease (second–third week), the virus can be intermittently detectable in nasopharyngeal swabs (9, 20).

Some authors reported a positive correlation between the severity of clinical conditions and upper respiratory tract viral load (25, 27).

Regarding the comparison of naso-and oropharyngeal swabs, the opinions are discordant: Wölfel et al. (23) state that no differences in viral loads or detection rates were revealed when comparing naso- and oropharyngeal swabs, while Zou et al. (25) and Yang et al. (26) noticed higher viral loads and detection rates in the nose swabs. Yu et al. (21), contrariwise, found a higher mean viral load in the throat (2,552 vs. 651 copies/test, *p* < 0.001).

#### Blood Specimens

Blood positivity rates reported among COVID-19 patients vary between 0 and 22% (10, 12, 14, 18–21, 23). Chen et al. affirm that the detection of viral RNA in the blood is a strong indicator of illness severity (10).

#### Feces Specimens and Anal Swabs

Stool content of viral RNA was detected in a great percentage of patients enrolled in various studies (11, 14, 19, 20, 23, 24). Wolfel et al. noted that the viral load in the stool seemed to reflect the sputum viral content (23).

Several authors therefore suppose an infection of the gastro-intestinal tract by the virus (11, 24), with its continuous elimination with the feces which has been reported to last from 1 to 12 days (24) and in some cases, viral RNA were detected in feces or anal swabs even after the respiratory tests became negative (11, 22, 24). Zhang et al. also report that during the first days of illness, the most positive swabs were the oral ones, whereas in the following days more and more anal swabs were positive, and oral ones negative (22).

#### Sputum Samples

Sputum samples appear to contain the maximum viral load (16, 21, 23), reaching the peak on the 5–6th day after symptoms onset (16) and remain positive for a maximum duration over time, compared to swabs of the upper respiratory tract (23). They also show one of the highest positivity rates (53.42–100%) among the tested samples (19, 21, 23, 24, 26), giving positive results for a long time, even when the pharyngeal samples are negative for the presence of the SARS-CoV-2 RNA (15), sometimes even after symptoms have ended (23). Some authors state that the sputum viral load seems to be significantly correlated to the pharyngeal one (12, 16).

#### Urine

All patients, except one in Kim's report (12) and four reported by Liu et al. (27) had negative viral detection in urine.

#### Saliva

Results on viral RNA detection in saliva are reported in two papers (17, 18). The detection rate in the initial samples is estimated to be around 90% (17, 18). The serial daily sampling revealed that the viral load was highest during the first week of symptoms and declined in the following days. On day 20 after symptoms onset, 33% of patients had viral RNA detected in the saliva specimens (18).

The main findings of the included studies are summarized in the Table 2.

**Table 2:** Main findings of the included studies.

	**Main findings in specimens**							**Notes**
**Author**	**Pharyngeal swabs**	**Blood/plasma/serum**	**Sputum**	**Feces**	**Urine**	**Anal swabs**	**Saliva**	
Chen et al. (10)		Positive in 6/57 (10,52%) of patients				Positive in 11/28 (39,28%) patients		Positive correlation of serum viral RNA with the disease severity supposed.
Chen et al. (11)				Positive in 28/40 (66.67%) patients	Positive in 0/10 (0%) patients			18/28 (64.29%) patients remained positive for viral RNA in feces for 7 (6–10) days after pharyngeal swabs turned negative
Lan et al. (9)								All patients, had 2 consecutive negative RT-PCR tests during recovering stage, returning to be positive 10–18 days later
Lescure et al. (14)	Maximum viral load in the first days of illness	Positive in 1/5 (20%) patients		Positive in 2/5 (40%) patients	All negative			
Liu et al. (27)					Positive in 4/6 (66,66%) patients			The viral load detected from respiratory tracts was positively linked to lung disease severity
Lo et al. (15)	9/10 (90%) positive at the first test			10/10 (100%) positive at the first test	Positive in 0/10 (0%) patients			
Kam et al. (13)	Positive in 2/2 (100%) patients	Positive only 1 day in 1 patient		1 positive value during illness course				
Kim et al. (12)	Positive in 2/2 (100%) patients	Few positive values during the illness course	Positive in 2/2 (100%)		Positive in 1/2 (50%) patient			
Pan et al. (16)	High viral load early after onset		High viral load early after onset					Viral loads of pharyngeal and sputum samples were significantly correlated
To et al. (17)							First specimens: positive in 91.66% of patients	
To et al. (18)		First specimen: positive in 22% of patients			All negative	First specimen positive in 27% of patients	First specimen: positive in 87%	
Wang et al. (19)	Positive in 126/398 (32%) of samples	Positive in 3/307 (1%) of samples	Positive in 75/104 (72%) of samples	Positive in 44/153 (29%) of samples	Positive 0/72 (0%) of samples			
Wölfel et al. (23)	Positive in 100% of cases on days 1–5	All negative	Positive in 100% of patients	Positive in 89% of patients	Positive in 0/9 (0%) patients			
Xiao et al. (24)			Positive 39/73 (53.42%) patients					17/39 (43.58%) patients remained positive in stool after showing negative in respiratory samples
Yang et al. (26)	Nasal/oral swabs positive in 73%/60% of cases in early stage.		Positive in 85% of samples in early stage					
Young et al. (20)	Positive in 100% Positive over 7 days in 83%	Positive in 1/12 (8.33%) patients		Positive in 4/8 (50%) patients	All negative			
Yu et al. (21)	Positive in 9/55 (16.4%) of nasal swabs, 50/134 (37.3%) of throat swabs	Positive in 0/4 (0%) samples	Positive in 77/116 (66.4%) samples		Positive in 0/14 (0%) samples			Analyzed with ddPCR
Zhang et al. (22)	More positive in early period					More positive in later period		
Zou et al. (25)	Viral load higher in patients with severe illness condition							

## Discussion

The pandemic spread of coronavirus infection SARS-CoV-2 forced many countries to take strong containment measures (33, 34). To avoid an uncontrolled broadcast of the disease, it is fundamental to understand the manner and timing of disease transmission. Then, a reliable test is needed to identify infected subjects, to take appropriate isolation measures for a period sufficient enough to avoid contagion of other individuals.

The reference method for testing positivity to SARS-CoV-2 infection is represented by the pharyngeal swab that is taken from the patient's nasopharynx or oropharynx and, through an RT-PCR analyzed for the presence of viral RNA (8). This method has been reasonably chosen, as it has already been used for other viruses affecting the airway tract, such as SARS-CoV (35). The wide use of such protocol is due to its multiple advantages. It is simple to perform, relatively inexpensive, and fast. However, as has emerged from recent studies, and confirmed by our cumulative analysis, the accuracy in the diagnosis of this swab seems to be excellent in the first phase of the disease, losing sensitivity in the following days (16, 20–23, 25). This can be linked to a reduction in the viral load present in the upper respiratory tracts starting from the second week of the disease (14, 16, 20, 23).

These data reveal two aspects to reflect on. The first one concerns the initial phase of the disease, that is, when the symptoms arise and the viral load in the upper airways is already almost at the peak, as suggested by several authors (14, 16, 23). This implies that many patients may be infectious for days before they show signs of disease. The second reflection concerns the terminal phase of the disease. In particular, attention should be paid to patients who test negative for the pharyngeal swab in the advanced stages of the disease, since Young et al. (20) and Lan et al. (9) show that the swab may be positive intermittently in this phase. Therefore, it is fundamental to understand whether the virus can be transmitted in this stage of disease. The presentation of the results of the RT-PCR analysis, however, remains only for a diagnostic purpose, without being able to provide indications on the contagiousness of the positive subject. Other methods like isolation and culture of the virus, are needed for this estimate (8).

For diagnostic proposes, it should be considered, as stated by Yu et al., that the performance of a droplet digital PCR (ddPCR) in SARS-CoV-2 detection may be significantly better compared to the traditional RT-PCR, especially for low viral loads (21).

In addition, according to some authors, there seems to be a difference between nasopharyngeal and oropharyngeal swabs (21, 25). In particular, one study with a high overall number of performed swabs (250 throat and 490 nasal swabs) state that the nasal swabs have a significantly higher positive rate than the oropharyngeal ones (73.3% vs. 60% in the first 7 days and 72.3% vs. 50.0% during the second week of illness) (26).

Among the investigated samples, saliva seems to be a promising specimen for detection of SARS-CoV-2 (17, 18). Authors found a positivity rate in the initial saliva samples of 87%, with a median viral load of 3.3 × 10^6^ copies per mL, values that seem to be similar to the pharyngeal swabs (ranging between 104 and 107 copies per mL) (16). The temporal course of the viral load in the saliva seems to follow that of the pharynx (18), even if it was not possible to refer data to the symptoms onset, but only to the hospitalization timing.

The sputum, seems to possess the highest positive rate among all the specimens (26), except for bronchoalveolar lavage fluid (BALF) (19, 26), and persists throughout the course of the disease (21, 23, 24, 26). The study investigating the active viral replication in the cells using sgRNA, found that the active replication of SARS-CoV-2 in the sputum samples persisted until days 10/11 of the illness, unlike pharyngeal swabs, where sgRNAs were no longer detectable at the end of the first week of symptoms (23). As suggested by Lo et al. (15) the sputum could be useful in the diagnosis of some suspected cases that are negative with repeated pharyngeal swabs.

Regarding the advanced stages of the disease, a fair rate of SARS-CoV-2 positivity was found in the stool of infected patients. In studies investigating the presence of viral RNA in the feces, more than half [and up to 90% reported by Lo et al. (15)] of the patients tested positive (11, 24). Furthermore, sometimes the fecal specimen remained positive, even after the pharyngeal specimen became negative (11, 22, 24). We do not know what implications this data has on the transmission or on the course of the disease, however, fecal examination should be considered to complement the diagnosis of COVID-19 patients.

The presence of viral RNA in the blood has also been investigated. However, few patients appear to have viremia during the course of the disease (14, 18, 19). Although, this event appears to be positively correlated with the severity of the symptoms (10).

No viral RNA was detected in breast milk (13), nor in vaginal fluids (28) of infected women.

An attempt was made in this overview to compare the Ct values of the main specimens that were found in the various studies during the course of the disease. Surely this result may be affected by a bias due to the difference in the methods and targets used in the various studies, even if there are universally accepted cut-offs (Ct-value < 40) that give us a reference in the interpretation of the results (8).

Another important aspect regarding the SARS-CoV-2 genome, and thanks to the availability of the newest sequencing methods and highly organized databases, several researchers are investigating genetic characteristics of the virus, subtype evolution, as well as geographic and temporal changes in the virus genome. Major attention has been focused on homoplasies, that is mutations that have emerged multiple times and may represent the sign of ongoing adaptation of the virus to the new human host. Several mutations in different regions of the viral genome have been found. These include sites in the Orf1ab region, Spike protein (36, 37), as well as the N gene (38). The implications of such mutations are not completely known. Some of them can be neutral (39), but it can be supposed that the changes in surface glycoprotein can influence the interaction between the virus and the host cell, as well as the anti-genicity of the virus (36, 40, 41).

A great part of knowledge about the genomic stability of SARS-CoV2 is still in evolving. It is still unclear if some sequence differences found in samples coming from different continents represent a temporal rather than a geographic signal. Further studies are needed to better define the behavior of the virus, in order to develop efficient treatments.

## Conclusions

A comprehensive approach of this overview was chosen in order to include as much data as possible in the final analysis, making it possible to analyze the data related to 889 patients, while all data reported the results differently. The results in the included studies were reported unevenly. Some were reports of a few patients, others presented data for many patients, but in a synthetic way. For this reason, the homogeneous data have been grouped together as far as possible and others treated discursively. However, some important conclusions emerged:

- the sputum, together with the bronchoalveolar lavage fluid, closely reflect the course of the infection;
- the pharyngeal swabs have a high accuracy in the initial phase of the disease, while their positivity rate drops suddenly in the following phases;
- viral RNA could be eliminated in the stool even for prolonged periods and their examination could supplement the pharyngeal swab.

Further studies with standardized protocols and an equally large number of samples for all types of specimens would be needed to draw more precise conclusions.

## Data Availability Statement

Publicly available datasets were analyzed in this study. The data can be found in papers cited in the References.

## Author Contributions

KZ and GP conceived the research. GT, KZ, and VC performed the literature search, analyzed the data, and wrote the article. LL supervised the research. All authors contributed to the article and approved the submitted version.

## Conflict of Interest

The authors declare that the research was conducted in the absence of any commercial or financial relationships that could be construed as a potential conflict of interest.
