# Supplementary material for: An Overview of the Temporal Shedding of SARS-CoV-2 RNA in Clinical Specimens
Source: Front Public Health. 2020 Aug 20;8:487. doi: 10.3389/fpubh.2020.00487 (PMC7468374; doi:10.3389/fpubh.2020.00487)
Supplement: Supplementary file 1 [file Data_Sheet_1.docx]

Supplementary materials:


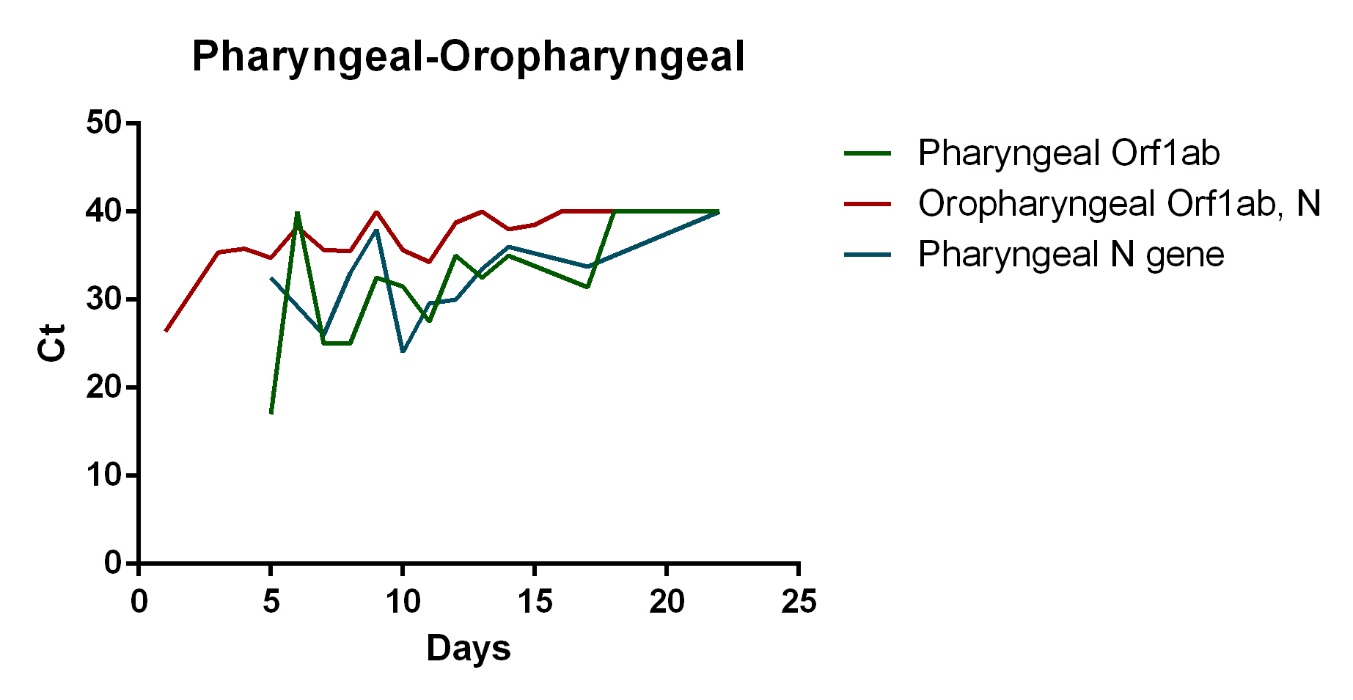


Figure 1: The time course of RT-PCR Ct values in Pharyngeal and oropharyngeal specimens represented separately for each molecular target.


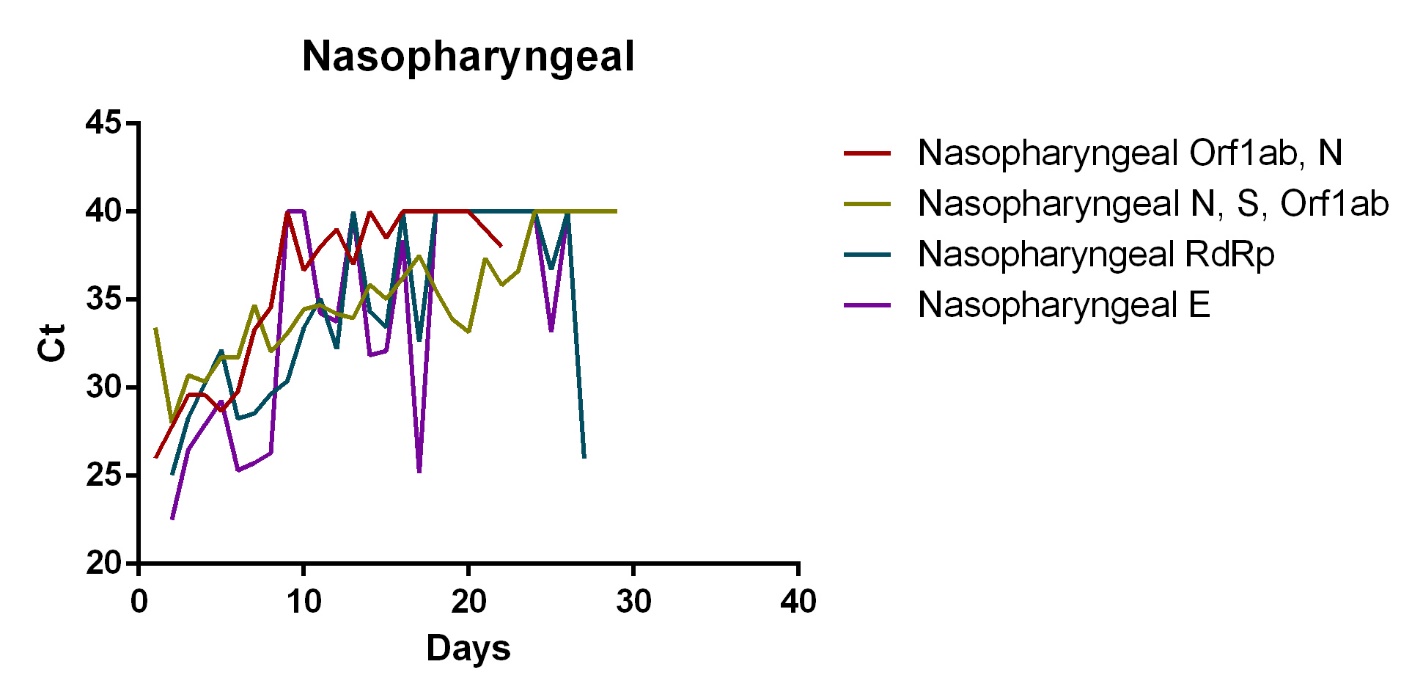


Figure 2: The time course of RT-PCR Ct values in Nasopharyngeal specimens represented separately for each molecular target.


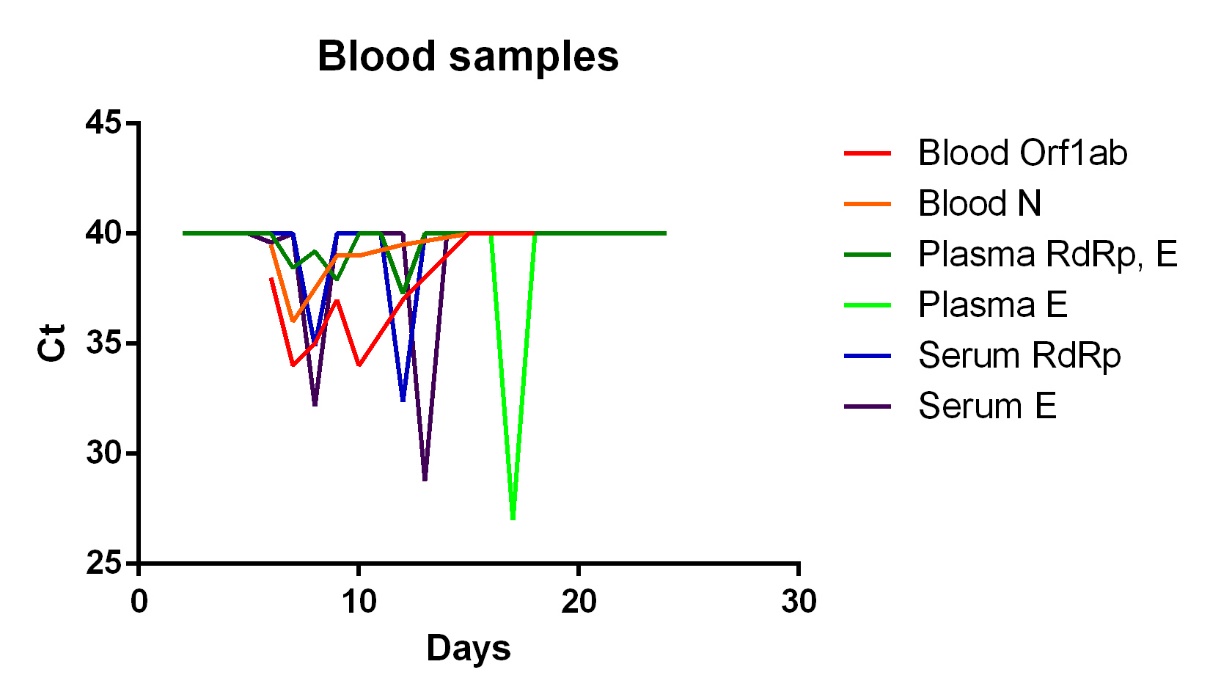


Figure 3: The time course of RT-PCR Ct values in Blood speciments, represented separately for each molecular target.


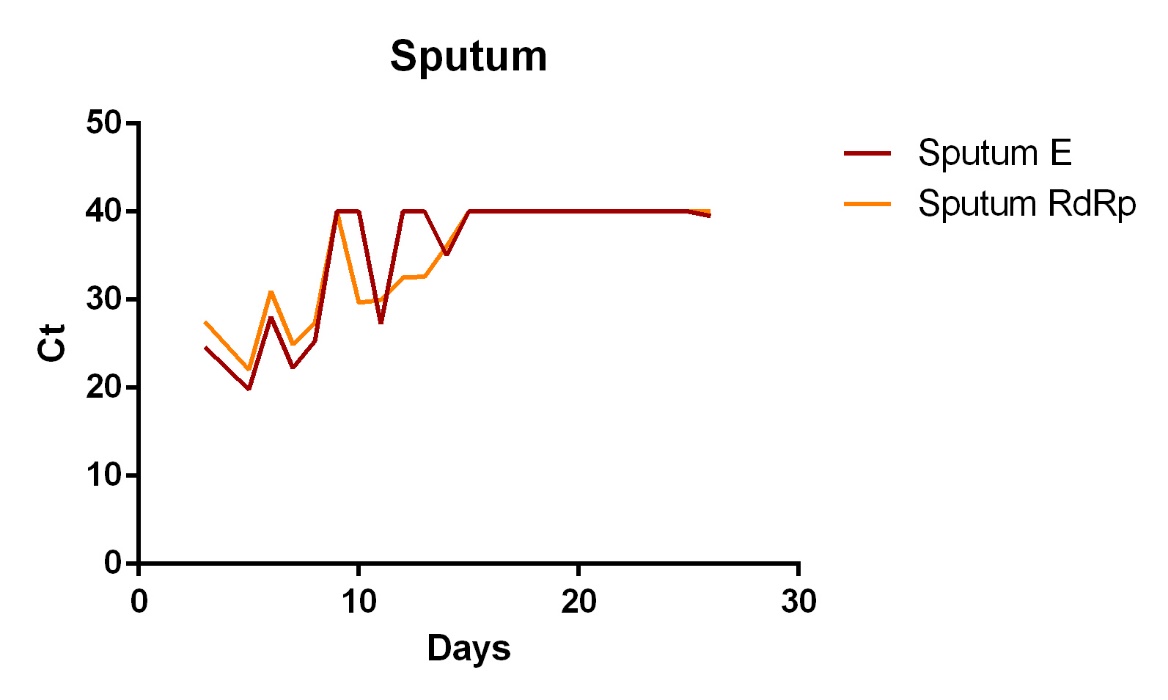


Figure 4: The time course of RT-PCR Ct values in Sputum represented separately for each molecular target.


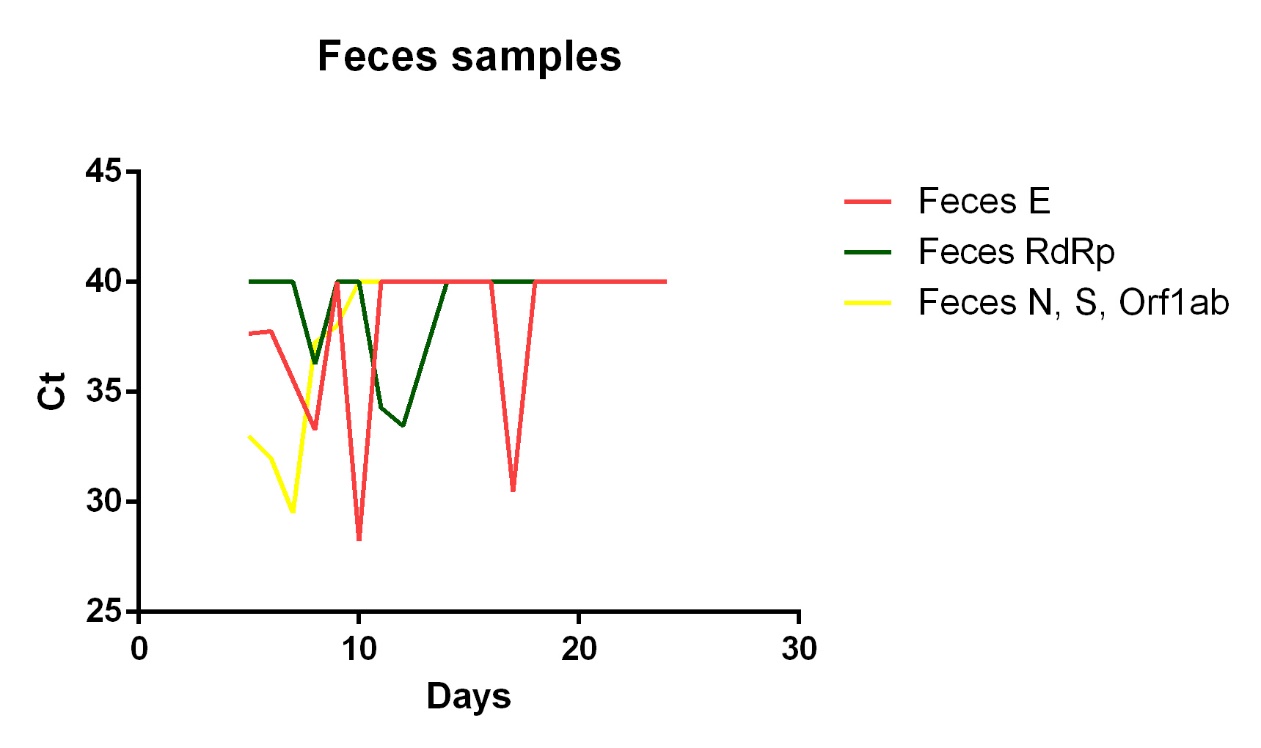


Figure 5: The time course of RT-PCR Ct values in Feces specimens, represented separately for each molecular target.

**Table: Characteristics of specimens and targets revealed in 51 patients included in the quantitative analysis.**

| **Specimen** | **Molecular target** | **Positive/Total number of tests (%)** | **Ct min (day)** |
| --- | --- | --- | --- |
| Pharyngeal swab | Orf1ab | 11/19 (57,89%) | 15 (d11) |
|  | N | 12/17 (70,58%) | 24 (d10) |
| Nasopharyngeal swab | N, S, Orf1ab | 151/216 (69,90%) | 20 (d4) |
|  | Orf1ab, N | 24/63 (38,09%) | 19 (d1) |
|  | RdRp | 15/27 (55,55%) | 25.05 (d2) |
|  | E | 14/27 (51,85%) | 22.5 (d2) |
| Oropharyngeal swab | Orf1ab, N | 31/66 (46%) | 19 (d1,d3) |
| Blood | Orf1ab | 8/15 (53,33%) | 32 (d8) |
|  | N | 8/13 (61,53%) | 36 (d7) |
|  | N, S, Orf1ab | 2/21 (9,52%) | 32.7 (d13) |
| Plasma | RdRp | 0/13 (0%) | - |
|  | E | 1/13 (7,69%) | 26.97 (d17) |
| Serum | RdRp | 2/24 (8,33%) | 32.37 (d12) |
|  | E | 3/24 (12,5%) | 28.76 (d13) |
| Feces | E | 6/20 (30%) | 28.2 (d10) |
|  | RdRp | 3/20 (15%) | 33.45 (d12) |
|  | N, S, Orf1ab | 7/21 (33,33%) | 26 (d5,d6) |
| Urine | RdRp | 1/23 (4,34%) | 29,62 (d12) |
|  | E | 3/23 (13,04%) | 30,12 (d9) |
|  | N, S, Orf1ab | 0/9 (0%) | - |
| Sputum | RdRp | 10/26 (38,46%) | 22.05 (d4) |
|  | E | 8/26 (30,76%) | 19.83 (d5) |
| Anal swabs | Orf1ab | 2/4 (50%) | 23 (d13) |
|  | N | 2/3 (66,67%) | 27 (d13) |
| Bronchoalveolar fluid | Orf1ab, N | 4/5 (80%) | 19.2 (d8) |

Positive/Total number of tests (%): percentage of positive specimens out of the total number.

Ct min: the lowest value of Cycle threshold observed; d: day of illness.
